# Supplementary material for: Peroxisomes form intralumenal vesicles with roles in fatty acid catabolism and protein compartmentalization in Arabidopsis
Source: Nat Commun. 2020 Dec 4;11:6221. doi: 10.1038/s41467-020-20099-y (PMC7718247; doi:10.1038/s41467-020-20099-y)
Supplement: Supplementary file 1 — Supplementary Figures [file 41467_2020_20099_MOESM1_ESM.pdf]

Supplementary Information

Peroxisomes form intralumenal vesicles with roles in fatty acid catabolism and protein compartmentalization in Arabidopsis; Zachary J. Wright and Bonnie Bartel; *Nature Communications*

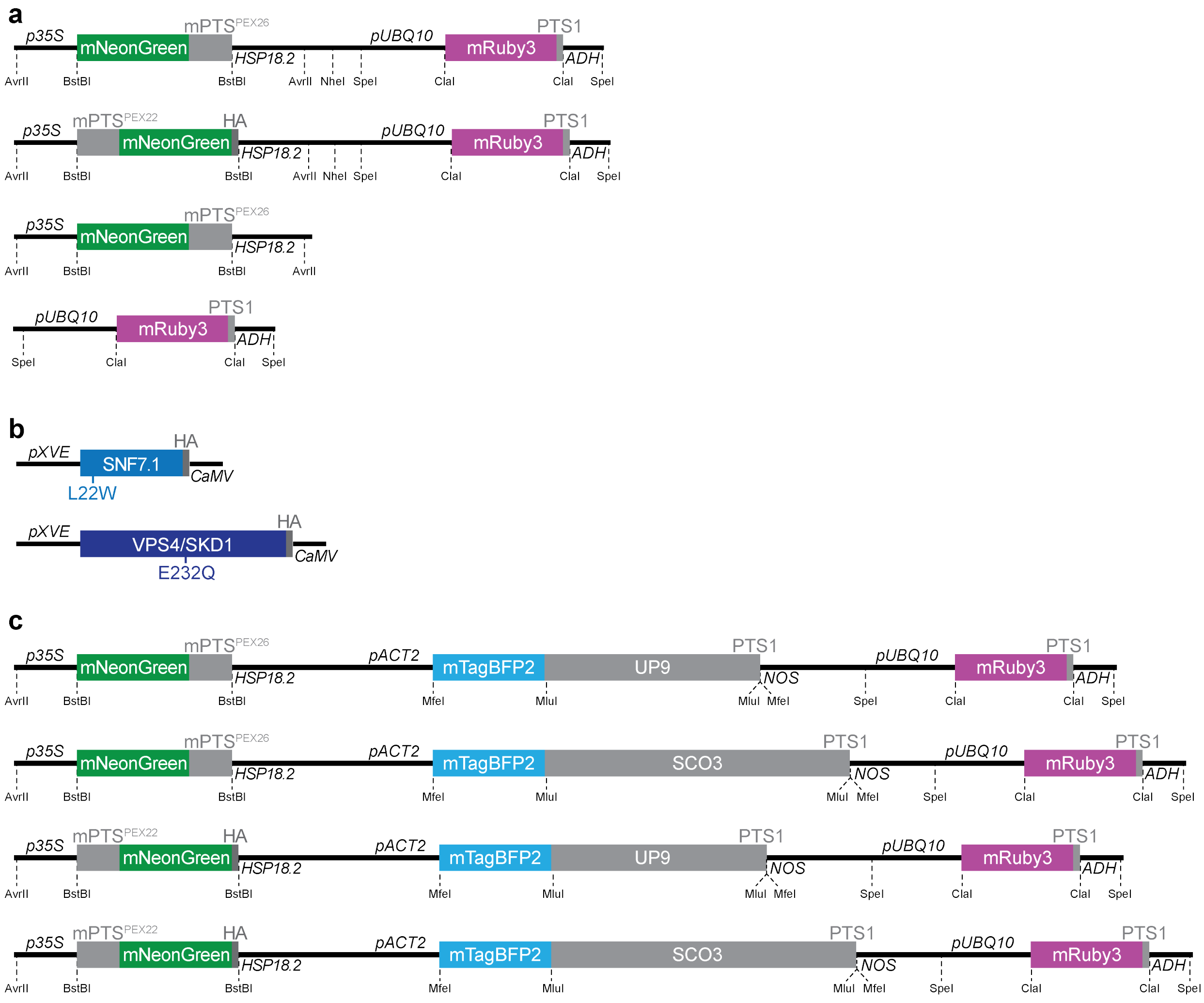

Supplementary Fig. 1. Diagrams of peroxisomal reporter constructs and dominant-negative ESCRT constructs. a, Dual and single reporters for the peroxisome membrane and lumen. Peroxisomal membrane reporters (mNG-mPTS^{PEX26} or mPTS^{PEX22}-mNG) are driven by the *35S* promoter and terminated by the *HSP18.2* terminator. The peroxisome lumen reporter (mRuby3-PTS1) was driven by the *UBQ10* promoter and terminated by the *ADH* terminator. **b**, Inducible dominant-negative ESCRT constructs. SNF7^{L22W} and VPS4^{E232Q} were C-terminally HA-tagged, driven by the β-estradiol inducible *pXVE* promoter system, and terminated by the *CaMV* terminator. **c**, Trifluorescent reporters with peroxisome membrane and lumen reporters from panel **a** and mTagBFP2 fused to the N-terminus of UP9 or SCO3 and driven by the *ACT2* promoter and terminated by the *NOS* terminator.

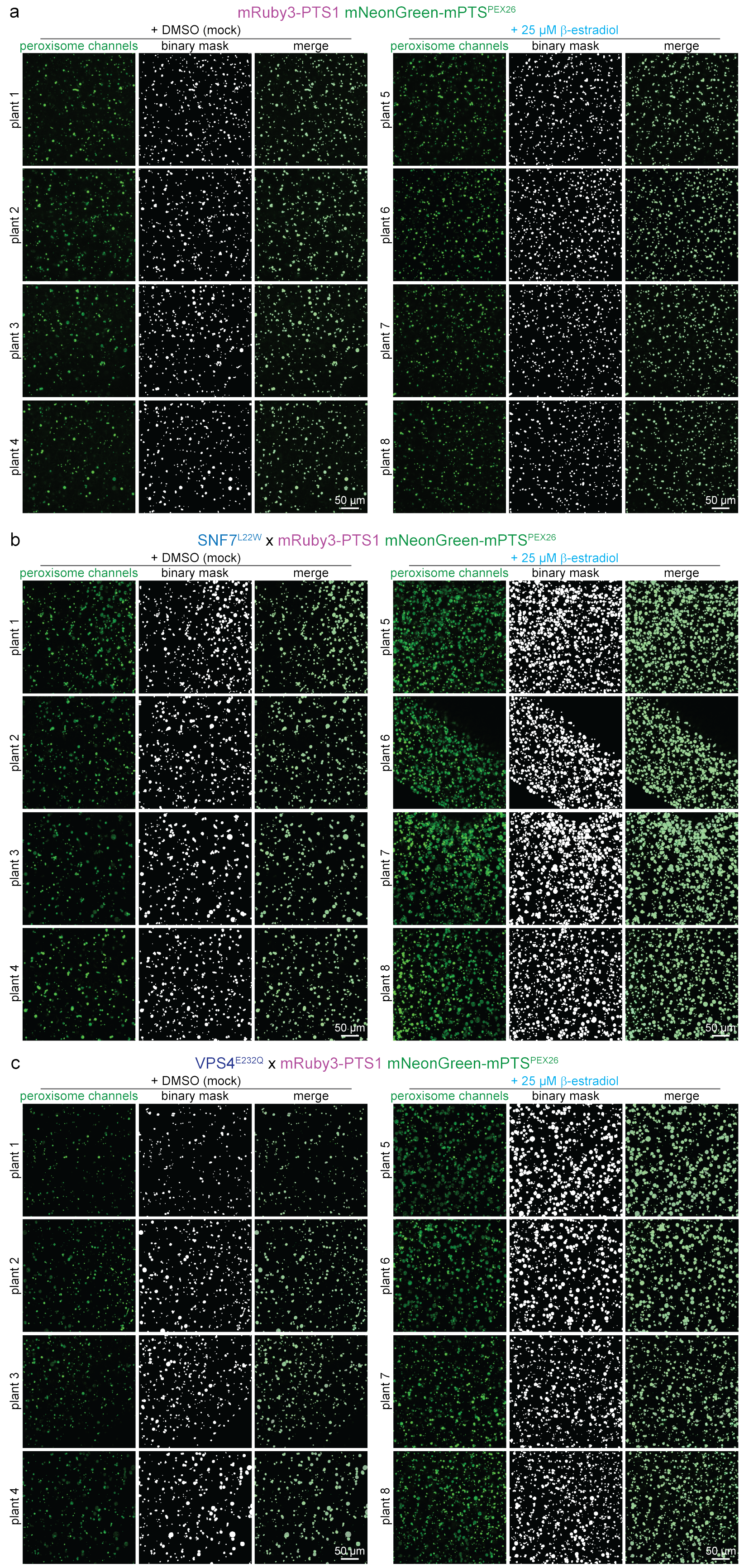

Supplementary Fig. 2. Images and binary masks used to quantify peroxisome size after dominant-negative ESCRT expression. 3D projections of peroxisome channels (mNeonGreen and mRuby3, green), binary masks used for diameter quantification (white), and peroxisome channels merged with masks from mPTS^{PEX26}/mRuby3-PTS1 (**a**) wild-type seedlings (**b**) SNF7^{L22W}, or (**c**) VPS4^{E232Q} F_4 seedlings grown for 5 days with DMSO (mock) or 25 µM β-estradiol.
