# Supplementary material for: Peroxisomes form intralumenal vesicles with roles in fatty acid catabolism and protein compartmentalization in Arabidopsis
Source: Nat Commun. 2020 Dec 4;11:6221. doi: 10.1038/s41467-020-20099-y (PMC7718247; doi:10.1038/s41467-020-20099-y)
Supplement: Supplementary file 3 — Descriptions of Additional Supplementary Files [file 41467_2020_20099_MOESM3_ESM.pdf]

Descriptions of Additional Supplementary Files

Supplementary Movie 1

Description: mPTSPEX26 membrane reporter reveals abundant inner membranes in peroxisomes. Imaging cotyledons of 4-day-old seedlings expressing mNG-mPTSPEX26 (green) and mRuby3-PTS1 (magenta) from the top of the epidermal cells to the beginning of the mesodermal cells reveals ILVs in peroxisomes. Images were acquired using a 1.04 µm interval with a 2.08 sec scan time. Scale bar is 20 µm.

Supplementary Movie 2

Description: mPTSPEX22 membrane reporter reveals abundant inner membranes in peroxisomes. Imaging cotyledons of 3-day-old seedlings expressing mPTSPEX22-mNG (green) and mRuby3-PTS1 (magenta) from the top of the epidermal cells to the beginning of the mesodermal cells reveals ILVs in peroxisomes. Images were acquired using a 0.25 µm interval with a 1.56 sec scan time. Scale bar is 20 µm.

Supplementary Movie 3

Description: Multivesicular mPTS-labeled structures are not endosomes. Imaging cotyledons of 4-day-old seedlings expressing mNGmPTSPEX26 (green) and stained with FM4-64 to mark the plasma membrane and endosomes (magenta) from the top of the epidermal cells to the beginning of the mesodermal cells. mNG-mPTSPEX26-labeled membranes lack FM4-64 and are distinct from endosomes. Images were acquired using a 0.38 µm interval with a 1.56 sec scan time. Scale bar is 20 µm.

Supplementary Movie 4

Description: Multivesicular mPTS-labeled structures are not autophagosomes. Imaging cotyledons of 4-day-old atg7-4 seedlings expressing mNG-mPTSPEX26 (green) and mRuby3-PTS1 (magenta) from the top of the epidermal cells to the beginning of the mesodermal cells reveals ILVs in peroxisomes. Images were acquired using a 1.04 µm interval with a 2.08 sec scan time. Scale bar is 20 µm.

Supplementary Movie 5

Description: Peroxisomal ILVs are visible with a lumenal reporter. Time-lapse imaging of hypocotyl cells of 3-day-old seedlings expressing the lumenal mRuby3-PTS1 (magenta) reporter show peroxisomal ILVs as holes in mRuby3-PTS1 signal that appear to float freely in the peroxisome lumen. Imaged were acquired with a 0.5 µm interval with a 0.76 sec scan time. Scale bar is 5 µm.

Supplementary Movie 6

Description: Peroxisomes decrease in size over time as ILVs form. 3D projections of cotyledons of 4-day-old seedlings expressing mNGmPTSPEX26 (green) and mRuby3-PTS1 (magenta) imaged at high magnification with a spinning disk confocal microscope every 15 minutes for 20 hours. Large peroxisomes decrease in size over time as they acquire ILVs. Scale bar is 10 µm. The region shown in Figure 2a is boxed.

Supplementary Movie 7

Description: Peroxisomes decrease in size over time as inner membrane density increases. 3D projections of cotyledons of 4-day-old seedlings expressing mNG-mPTSPEX26 (green) and mRuby3-PTS1 (magenta) imaged at low magnification with a scanning confocal microscope every 15 minutes for 9 hours. Large peroxisomes decrease in size over time while small peroxisomes stay small. Scale bar is 20 µm. The region shown in Figure 2b is boxed.

Supplementary Movie 8

Description: Peroxisomal ILVs do not contain engulfed lipid droplets. Imaging cotyledons of 4-day-old wild-type seedlings expressing mNG-mPTSPEX26 (green) and stained with MDH to detect lipid droplets (magenta) from the top of the epidermal cells to the beginning of the mesodermal cells. Images were acquired using a 0.1 µm interval with a 0.52 sec scan time. Scale bar is 5 µm.

Supplementary Movie 9

Description: Peroxisomal ILVs are apposed to lipid droplets. Imaging cotyledons of 4-day-old acx2-2 seedlings expressing mNG-mPTSPEX26 (green) and stained with MDH to detect lipid droplets (magenta) from the top of the epidermal cells to the beginning of the mesodermal cells. Images were acquired using a 0.1 µm interval with a 0.76 sec scan time. Scale bar is 5 µm. Supplementary Movie 10. Peroxisomal ILVs may have distinct membrane protein domains. Imaging cotyledon epidermal cells of 4-day-old seedlings expressing mNG-mPTSPEX26 (green) and mRuby3-PTS1 (magenta) reveals
