# Supplementary material for: Peroxisomes form intralumenal vesicles with roles in fatty acid catabolism and protein compartmentalization in Arabidopsis
Source: Nat Commun. 2020 Dec 4;11:6221. doi: 10.1038/s41467-020-20099-y (PMC7718247; doi:10.1038/s41467-020-20099-y)
Supplement: Supplementary file 14 — Reporting Summary [file 41467_2020_20099_MOESM14_ESM.pdf]

nature research

Corresponding author(s): Bonnie Bartel

Last updated by author(s): Nov 7, 2020

Reporting Summary

Nature Research wishes to improve the reproducibility of the work that we publish. This form provides structure for consistency and transparency in reporting. For further information on Nature Research policies, see our Editorial Policies and the Editorial Policy Checklist.

Statistics

For all statistical analyses, confirm that the following items are present in the figure legend, table legend, main text, or Methods section.

n/a	Confirmed	
[ ]	[x]	The exact sample size (*n*) for each experimental group/condition, given as a discrete number and unit of measurement
[ ]	[x]	A statement on whether measurements were taken from distinct samples or whether the same sample was measured repeatedly
[ ]	[x]	The statistical test(s) used AND whether they are one- or two-sided<br>*Only common tests should be described solely by name; describe more complex techniques in the Methods section.*
[x]	[ ]	A description of all covariates tested
[x]	[ ]	A description of any assumptions or corrections, such as tests of normality and adjustment for multiple comparisons
[ ]	[x]	A full description of the statistical parameters including central tendency (e.g. means) or other basic estimates (e.g. regression coefficient) AND variation (e.g. standard deviation) or associated estimates of uncertainty (e.g. confidence intervals)
[ ]	[x]	For null hypothesis testing, the test statistic (e.g. *F*, *t*, *r*) with confidence intervals, effect sizes, degrees of freedom and *P* value noted<br>*Give P values as exact values whenever suitable.*
[x]	[ ]	For Bayesian analysis, information on the choice of priors and Markov chain Monte Carlo settings
[x]	[ ]	For hierarchical and complex designs, identification of the appropriate level for tests and full reporting of outcomes
[x]	[ ]	Estimates of effect sizes (e.g. Cohen's *d*, Pearson's *r*), indicating how they were calculated

Our web collection on statistics for biologists contains articles on many of the points above.

Software and code

Policy information about availability of computer code

Data collection	ZEN 2010 version 6.0.0.485 (for Zeiss 710) and ZEN 2.6 version 2.6.76.00 (for Zeiss 800) (Carl Zeiss - https://www.zeiss.com/microscopy/us/products/microscope-software/zen.html); Andor iQ software, version 3.4 (https://andor.oxinst.com/products/iq-live-cell-imaging-software)
Data analysis	ImageJ/FIJI (version 2.0.0), Ilastik (Version 1.3.2), R (Version 3.5.3), ggplot2 (version 3.3.2) Adobe Illustrator (version 24.2.3), and Microsoft Excel (Version 2010)

For manuscripts utilizing custom algorithms or software that are central to the research but not yet described in published literature, software must be made available to editors and reviewers. We strongly encourage code deposition in a community repository (e.g. GitHub). See the Nature Research guidelines for submitting code & software for further information.

Data

Policy information about availability of data

All manuscripts must include a data availability statement. This statement should provide the following information, where applicable:

- Accession codes, unique identifiers, or web links for publicly available datasets
- A list of figures that have associated raw data
- A description of any restrictions on data availability

The data that support the findings of this study are available in the main text or supplementary information. Source data for Fig. 2c, 3b, 3d, 4b, and 5b are provided with the paper.

Field-specific reporting

Please select the one below that is the best fit for your research. If you are not sure, read the appropriate sections before making your selection.

[x] Life sciences [ ] Behavioural & social sciences [ ] Ecological, evolutionary & environmental sciences

For a reference copy of the document with all sections, see nature.com/documents/nr-reporting-summary-flat.pdf

Life sciences study design

All studies must disclose on these points even when the disclosure is negative.

Sample size	No sample size calculations were performed. Sample sizes for immunoblots were chosen based on the amount of protein that can provide reliable detection signals on immunoblots as previously determined (e.g., Kao & Bartel, 2015, BMC Plant Biol. 15, 224). For experiments quantifying organelle size, images were collected from 2 or 3 cotyledons (as noted in methods) from different plants and all peroxisomes or lipid droplets in each image were measured.
Data exclusions	No data were excluded from the analyses.
Replication	All experiments were replicated at least twice and all attempts at replication were successful. For all fluorescent reporter lines used, lines from at least two independent insertions were imaged and a representative line was used for all experiments.
Randomization	Randomization is not relevant to this study. Age-matched seedlings carrying various transgenes were imaged or collected. Transgenes imaged in different genotypes (e.g., wild type and atg7) were obtained via crossing to avoid variation based on transgene insertion position. Other covariates were controlled by ensuring that plants used for comparison were of similar developmental stage.
Blinding	Blinding was not performed. For the microscopy experiments, blinding was not possible because the different transgenes conferred distinct fluorescent localization patterns (e.g., Fig. 1) or the effect was too apparent (e.g., Fig. 3 and Fig. 5). For the immunoblotting experiment, blinding was not compatible with having a meaningful lane order.

Reporting for specific materials, systems and methods

We require information from authors about some types of materials, experimental systems and methods used in many studies. Here, indicate whether each material, system or method listed is relevant to your study. If you are not sure if a list item applies to your research, read the appropriate section before selecting a response.

Materials & experimental systems

n/a	Involved in the study	
[ ]	[x]	Antibodies
[x]	[ ]	Eukaryotic cell lines
[x]	[ ]	Palaeontology and archaeology
[x]	[ ]	Animals and other organisms
[x]	[ ]	Human research participants
[x]	[ ]	Clinical data
[x]	[ ]	Dual use research of concern

Methods

n/a	Involved in the study	
[x]	[ ]	ChIP-seq
[x]	[ ]	Flow cytometry
[x]	[ ]	MRI-based neuroimaging

Antibodies

Antibodies used	Primary antibodies used were: rabbit anti-PMDH2 (Pracharoenwattana et al., 2007), rat anti-HA (Roche clone 3F10, Sigma 11867423001), and mouse anti-HSC70 (Stressgen SPA-817). Horseradish peroxidase-conjugated secondary antibodies were used: goat anti-rabbit IgG (GenScript A00098), goat anti-rat IgG (Invitrogen A10549 , lot number 1835025), and goat anti-mouse IgG (GenScript A00160).
Validation	The PMDH2 antibody was validated by the authors of the original study Pracharoenwattana et al., 2007 by using immunoblotting of extracts from a pmdh1 pmdh2 double mutant. The HA antibody does not detect proteins in immunoblots of Arabidopsis extracts lacking HA-tagged recombinant proteins (Figure 3b). The HSC70 antibody is a monoclonal antibody raised against spinach HSC70 that detects a single band on immunoblots of Arabidopsis seedling extracts (Figure 3b).
